# How Teichoic Acids Could Support a Periplasm in Gram-Positive Bacteria, and Let Cell Division Cheat Turgor Pressure

**DOI:** 10.3389/fmicb.2021.664704

**Published:** 2021-05-10

**Authors:** Harold P. Erickson

**Affiliations:** Department of Cell Biology, Duke University Medical Center, Durham, NC, United States

**Keywords:** periplasm, turgor pressure, teichoic acids, cartilage, plasmolysis, cryo-electron microscopy, peptidoglycan, FtsZ

## Abstract

The cytoplasm of bacteria is maintained at a higher osmolality than the growth medium, which generates a turgor pressure. The cell membrane (CM) cannot support a large turgor, so there are two possibilities for transferring the pressure to the peptidoglycan cell wall (PGW): (1) the CM could be pressed directly against the PGW, or (2) the CM could be separated from the PGW by a periplasmic space that is isoosmotic with the cytoplasm. There is strong evidence for gram-negative bacteria that a periplasm exists and is isoosmotic with the cytoplasm. No comparable studies have been done for gram-positive bacteria. Here I suggest that a periplasmic space is probably essential in order for the periplasmic proteins to function, including especially the PBPs that remodel the peptidoglycan wall. I then present a semi-quantitative analysis of how teichoic acids could support a periplasm that is isoosmotic with the cytoplasm. The fixed anionic charge density of teichoic acids in the periplasm is ∼0.5 M, which would bring in ∼0.5 M Na^+^ neutralizing ions. This approximately balances the excess osmolality of the cytoplasm that would produce a turgor pressure of 19 atm. The 0.5 M fixed charge density is similar to that of proteoglycans in articular cartilage, suggesting a comparability ability to support pressure. An isoosmotic periplasm would be especially important for cell division, since it would allow CM constriction and PGW synthesis to avoid turgor pressure.

## Introduction

Bacterial cytoplasm has a high concentration of proteins and nucleic acids, plus their neutralizing counterions and various small molecule osmolytes. The higher osmolality of the cytoplasm relative to the outside growth medium causes it to generate a turgor pressure on the cell envelope. An important question is whether constriction of the cell envelope at cell division needs to overcome the turgor pressure. I have previously argued that it does not, based on compelling evidence from gram-negative bacteria (Erickson, 2017). Here I extend this argument with an emphasis on gram-positive bacteria.

The envelope of gram-negative bacteria comprises an inner cytoplasmic membrane (CM), a peptidoglycan wall (PGW) and an outer membrane (OM). The OM is closely attached to the PGW by multiple covalent crosslinks, so the OM and PGW operate as a functional unit [see diagrams in Erickson (2017)]. The envelope of gram-positive bacteria has a CM and PGW and is lacking an OM. We will define the periplasm as the space between the outer face of the CM and the inner face of the PGW. Some authors have defined the periplasm as the space between the inner and outer membranes for gram-negative bacteria, but our definition provides consistency for gram-positive bacteria.

There are two possibilities for the state of the periplasm. In case 1 the CM is pressed against the PGW by cytoplasmic turgor pressure, and there is essentially no periplasmic space. In Case 2 the CM is separated from the PGW by a periplasmic space. I will first present a general argument that a periplasmic space is necessary for periplasmic proteins, in particular PGW remodeling enzymes, to function. I will review the abundant evidence that gram-negative bacteria do have a periplasm, and that the periplasm is isoosmotic with the cytoplasm. Evidence for a periplasm in gram-positive bacteria is more limited, but I will argue that a periplasm exists here also. Qualitative and quantitative analyses will suggest how teichoic acids could support and maintain this periplasmic space in gram-positive bacteria. I will conclude that the periplasm of both gram-negative and gram-positive bacteria are likely isoosmotic with the cytoplasm, which means that cytokinesis does not need to generate a force to overcome turgor pressure.

### A Periplasmic Space Is Needed for the Peptidoglycan Synthesis Machinery

Matias and Beveridge (2005) briefly proposed a general argument for why a periplasm is needed: “It is probable that PBPs require a certain amount of free space within the periplasm to catalyze the development of new wall fabric.” This argument needs reemphasis and elaboration.

**FIGURE 1:**
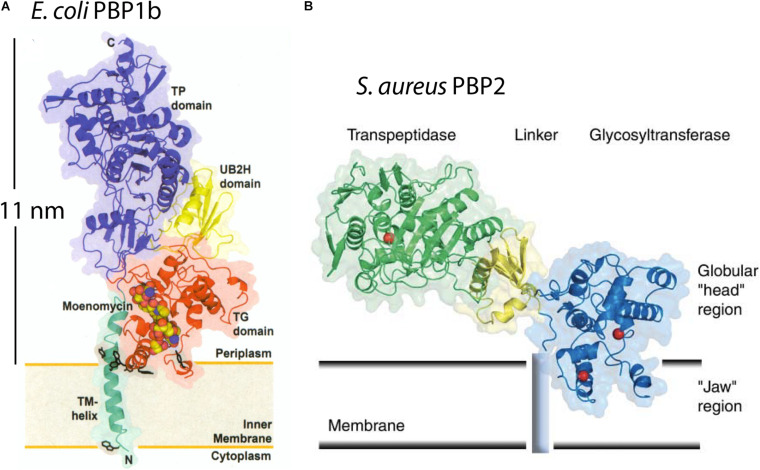
The x-ray structures are shown for *E. coli* PBP1b **(A)** and *S. aureus* PBP2 **(B)**. They project 11 nm or 6 nm above the CM in the interpretation of the authors (Lovering et al., 2007; Sung et al., 2009). This would require a periplasmic space of at least this width. Reprinted with permission from the referenced publications.

The *Escherichia coli* PBP1b is an elongated molecule 11.5 nm long, with its transglycosylase domain near its transmembrane attachment and its transpeptidase domain at the other end (Figure 1A; Sung et al., 2009). The perpendicular arrangement to the membrane was supported by the arrangement of the transmembrane helix, which was present in this crystal structure. PBP1b would thus span the 11 nm periplasmic space of *E. coli* and have its transpeptidase activity near the PGW. A structure of *Staphylococcus aureus* PBP2 showed a similar arrangement of the transglycosylase domain at the membrane and the transpeptidase domain about 10 nm distal (Figure 1B) (Lovering et al., 2007). These authors drew the molecule tilted with respect to the membrane, but this would still require a substantial periplasmic space. Even in the most extreme case, if the PBP were flattened against the CM, its thickness of 4–5 nm would necessitate a periplasm at least that thick.

We can estimate the force that a 20 atm turgor pressure would generate on a protein molecule. 20 atm is 2 × 10^6^ N/m^2^ = 2 pN/nm^2^. The tip of a PBP is about 2 × 2 nm, giving a total force of 8 pN pressing on the PBP. This is the same magnitude as the ∼5 pN force that stalls a kinesin or myosin motor molecule. If the PBP had to support a 20 atm turgor it would have to operate against the maximum force that can be generated by motor molecules.

An equally important consideration is the stereochemistry. In the extreme case where the PBP is squashed against the PGW, the transpeptidase domain would have to arrange the entrance of the peptides from two adjacent glycan strands, then form the peptide crosslink and release the product under this pressure. The transglycosidase would face similar difficulties. No structural study has addressed how a PBP might be able to function if it were flattened and squashed between the PGW and CM.

A final important point is that the diffusion of transmembrane proteins is similar in gram-negative and gram-positive bacteria. In *E. coli* the diffusion coefficient, D, ranged from 0.1 to 0.2 μm^2^s^–1^ for proteins with a transmembrane radius up to 2.5 nm (Oswald et al., 2016). In *Bacillus subtilis* D ranged from 0.2 to 0.5 μm^2^s^–1^ for proteins with 2–12 transmembrane segments (Lucena et al., 2018). Particularly relevant to cell division, the transpeptidases PBP3 (FtsI) in *E. coli*, and PBP2b in *B. subtilis* had virtually identical diffusion coefficients of 0.041 and 0.038 μm^2^ s^–1^ (McCausland et al., 2021). These values are lower than ranges quoted above, perhaps because the tall PBPs are interacting with the PGW. If the gram-positive CM were pressed against the PGW by turgor, one would expect the diffusion of transmembrane proteins, especially those with a bulky periplasmic domain, to be slowed almost to zero. The approximately equal diffusion coefficients measured in gram-negative and gram-positive bacteria argues for an equivalent periplasm in both.

### Evidence That the Periplasm of Gram-Negative Bacteria Is Isoosmotic With the Cytoplasm

Two studies have used radiotracers to measure the volume of the periplasm and cytoplasm in gram-negative bacteria, and how these volumes responded to osmotic shock. They provided compelling evidence that a periplasm exists and is isoosmotic with the cytoplasm. CryoEM has provided images of the periplasm consistent with these volume measurements.

The first study was from Stock et al. (1977). They labeled bacterial cell cultures with three molecules to differentiate the spaces in a bacterial pellet. ^3^H H_2_O, which permeates all spaces including the cytoplasm, was coupled with either ^14^C inulin, which is excluded by the OM and labels only the extracellular space, or ^14^C sucrose, which permeates extracellular space and the periplasm but not the cytoplasm. They found that the “periplasmic volume” (which here would include the space from the IM to the OM) of *E. coli* and *Salmonella typhimurium* was 20–40% of the total cell volume when growing under static osmolal conditions. When bacteria were exposed to an osmotic shock of sucrose, which crosses the OM but not the CM, the periplasm increased in volume as the cytoplasm leaked water and contracted. They concluded that the “cytoplasmic membrane is flexible and unable to support a pressure gradient…Under all conditions the periplasm and cytoplasm remained isoosmotic” (Stock et al., 1977).

Cayley et al. (2000) expanded the study to measure effects of osmotic shock on cells initially in low to high osmolal medium. For *E. coli* cells in low osmolal medium, the “periplasm” (here also from IM to OM) was 13% of the total cell volume. Upon osmotic shock of 1 M NaCl, which like sucrose crosses the OM but not the CM, the periplasm increased to 50% of the total cell volume. They concluded that “the periplasm and cytoplasm are isoosmotic, and that *E. coli* maintains turgor pressure across the cell wall and not across the cytoplasmic membrane” (Cayley et al., 2000). Cayley et al. (2000) is now the definitive study of osmolality of the periplasm in *E. coli* and gram-negative bacteria in general.

The osmolality of the gram-negative periplasm is thought to be generated by membrane-derived oligosaccharides, which were recently renamed osmoregulated periplasmic glucans (OPGs) (Bontemps-Gallo et al., 2017). OPGs are anionic glucose oligomers with an average charge of –5 and a size of ∼2,300 Da, which is too large to pass through the small pores of the OM-PGW layer (Kennedy, 1982; Miller et al., 1986; Cayley et al., 2000). The OPGs and their neutralizing cations generate an osmolality that matches that of the cytoplasm. Upon osmotic shock the periplasm expands, and this expansion persists for 30 min or more. However, if cells are maintained in a high osmolal medium they down-regulate synthesis of OPGs (Kennedy, 1982; Miller et al., 1986; Cayley et al., 2000). The periplasm then shrinks and the cytoplasm expands. Recent work suggests that there may be additional osmoregulatory mechanisms, since mutants defective in OPGs can survive; see (Bontemps-Gallo et al., 2017) for a comprehensive review.

**FIGURE 2:**
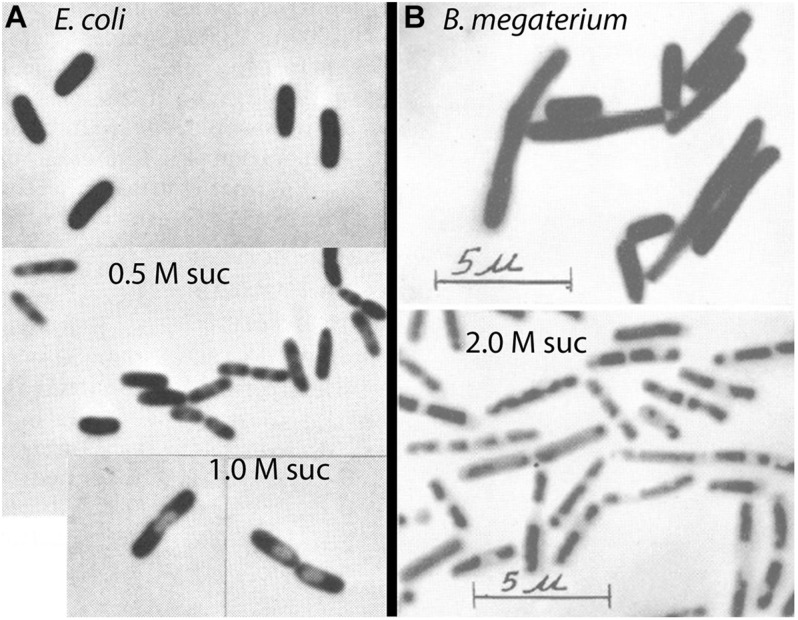
Phase contrast images of phase-light plasmolysis spaces induced by sucrose shock. **(A)** *E. coli* undergoes plasmolysis at 0.5 – 1.0 M sucrose (Scheie and Rehberg, 1972). **(B)** Gram-positive bacteria require much higher osmotic shock to produce visible plasmolysis (Weibull, 1965). Reprinted from the indicated references with permission.

Well before the study of Stock et al. (1977), the response of gram-negative bacteria to osmotic shock had been observed by phase contrast light microscopy. The response was termed “plasmolysis” and it involved the formation of one or two phase-light bubbles, usually located at the poles of the bacterium (Figure 2A). These “plasmolysis spaces” were understood to be expansions of the periplasm. Plasmolysis spaces in *E. coli* have been imaged more precisely in two recent studies. Sochacki et al. (2011) used periplasmic GFP to directly image the periplasm and its expansion upon osmotic shock. An elegant study of Pilizota and Shaevitz (2013) used super-resolution light microscopy to image the outer membrane and the cytoplasmic volume of *E. coli*, and thereby reconstruct the periplasm. Both studies found plasmolysis spaces at the poles of *E. coli* in response to osmotic shock of 0.3 M sucrose. We will discuss below that *B. subtilis* required much higher osmotic shock to produce visible plasmolysis (Figure 2B).

### Turgor Pressure of *E. coli* Is Highly Dependent on External Osmolality

Cayley et al. (2000) are widely referenced for determining the turgor pressure of *E. coli* to be ∼3 atm. A later study by Deng et al. (2011) reported a turgor pressure of only 0.3 atm, and this is sometimes referenced as questioning the 3 atm of Cayley et al. (2000). However, the 3 atm turgor in the Cayley et al. (2000) study applied only to growth in media of very low osmolality. They actually explored a full range of growth media (Table 1) and found that at higher osmolality the turgor dropped to the range later reported by Deng et al. (2011). It should be emphasized that normal growth media are in this higher osmolal range, where turgor pressure of *E. coli* is so small that it can hardly be measured.

**TABLE 1:** Turgor pressure of *E. coli* as a function of osmolality of growth medium.

**Osm of growth medium**	**ΔΠ atm**	**References**
0.03	3.1 ± 0.4	Cayley et al., 2000
0.10	1.5 ± 0.3	Cayley et al., 2000
0.28	0.7 ± 1.1	Cayley et al., 2000
0.8	<0.5	Cayley et al., 2000
0.44	0.3	Deng et al., 2011

### Existence of a Periplasm in Gram-Positive Bacteria Is Controversial

**FIGURE 3:**
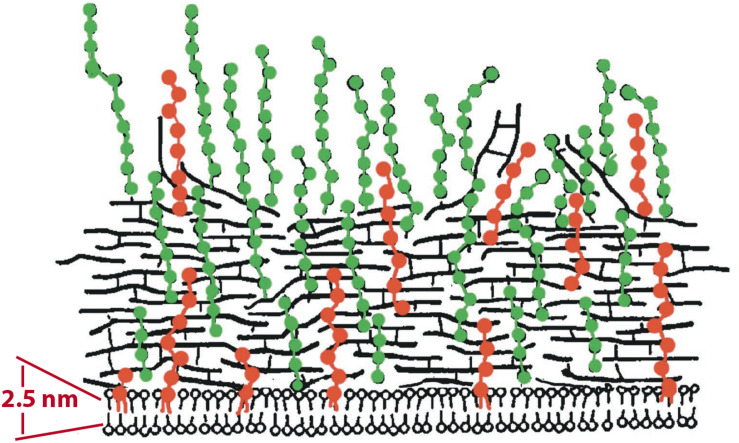
An illustration of the gram-positive cell envelope showing a modern view of the periplasm. Neuhaus and Baddiley (2003) drew the CM pressed against the PGW, leaving essentially no periplasmic space. Horizontal black lines represent PG; Teichoic acids are green (WTA) and red (LTA). An earlier illustration of Umeda et al. (1992), which was modified to generate this figure, showed a small periplasmic space separating the CM and PGW. Reprinted from Neuhaus and Baddiley (2003) with permission.

As detailed above, volume measurements using radiotracers have provided compelling evidence for a periplasmic space in *E. coli.* Remarkably, comparable measurements for gram-positive bacteria are almost completely lacking. There is currently a widespread belief that gram-positive bacteria don’t have a periplasm. This is illustrated in a figure from a modern review, where the CM is drawn pressed against the PGW (Figure 3) (Neuhaus and Baddiley, 2003). A much earlier diagram, which was revised to produce Figure 3, showed a small but distinct periplasmic space, based on early EM images (Umeda et al., 1992). In their more recent adaptation Neuhaus and Baddiley eliminated the periplasm, in keeping with the widespread belief. There is no room here for a PBP - even a bent-over PBP (Figure 1B) would project half-way through the indicated PGW.

Plasmolysis is the term used for the expansion of the periplasm in response to osmotic shock. As discussed above, this was easily visualized by phase contrast microscopy as formation of plasmolysis spaces in *E. coli*. In contrast, it has long been thought that gram-positive bacteria cannot be plasmolyzed. Weibull commented in 1955 “*B. megaterium* is not plasmolysable according to Fisher, as has been confirmed by the author” (Weibull, 1955). Ten years later he amended this and suggested “that most bacteria can be plasmolyzed, but that media of a very high osmotic pressure are required to effect plasmolysis in gram-positive organisms” (Weibull, 1965). By “high osmotic pressure” he meant greater than 1 M sucrose. His image of plasmolyzed *B. megaterium* is shown in Figure 2B.

**FIGURE 4:**
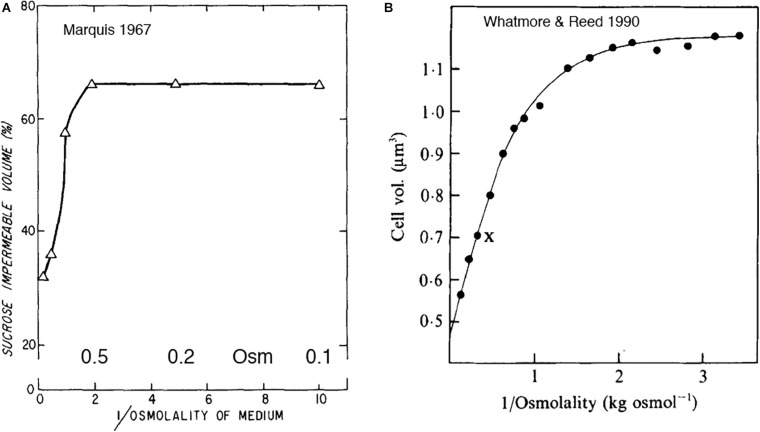
**(A)** An early study of Marquis (1967) showed shrinkage of gram-positive cytoplasm in response to osmotic shock, but only above 1 M sucrose. **(B)** Whatmore and Reed (1990) measured the response of cell volume to sucrose shock. The cell volume they report may be primarily the cytoplasmic volume. As in 4A, cytoplasmic volume decreased only above 1 M. Note that both graphs plot reciprocal osmolality on the x axis. Reprinted from the referenced works with permission.

One early study of plasmolysis of *B. megaterium*, a gram-positive bacterium, used a radiotracer technique similar to that of Stock et al. (1977) and Cayley et al. (2000). This study by Marquis (1967) actually preceded the Stock study by 10 years. Marquis used high molecular weight dextran, which cannot penetrate the PGW, to measure the total volume of the cells in a bacterial pellet, and radio-labeled sucrose, which can penetrate the PGW but not the CM, to measure the volume of cytoplasm. The cytoplasmic volume was 66% of the total cell volume for control cells and remained at this level for sucrose shocks of 0.1 to 0.5 Osm (Figure 4A). Cytoplasmic volume dropped to 57% at 1 Osm, and to ∼35% at very high osmolality. Interestingly, the data of Marquis showed no change in cytoplasmic volume for shocks of 0.1 to 0.5 Osm, and only a small drop at 1 Osm. Substantial shrinkage of the cytoplasm was only seen above 1 M sucrose, consistent with the light microscopy observations of Weibull (1965) (Figure 2B).

Whatmore and Reed (1990) reported a similar curve for sucrose shock of *B. subtilis* (Figure 4B). Their article is mostly referenced for determining the turgor pressure of *B. subtilis* to be 19 atm. Their data are also relevant to the question of plasmolysis. They used a “C1000 channelizer” to measure cell volume. This assay was suggested to report cell volume, but it may be reporting primarily cytoplasmic volume. They showed no change in cytoplasmic volume for sucrose shocks of 0.2 to 0.5 Osm, and decreasing volume for 1 Osm and above.

Whatmore and Reed (1990) analyzed the data in Figure 4B as a Boyle-van’t Hoff plot, and calculated a turgor pressure of 19 atm for growth of *B. subtilis* in media of 0.27 Osm. This is almost two orders of magnitude higher than *E. coli* growing at 0.3 Osm, so it is important to know if this extends to other gram-positive bacteria. The Poolman lab has recently done a similar analysis of cell volume vs osmotic shock for two species. *Lactococcus lactis* had a turgor of 19 atm (Mika et al., 2014) and *Listeria monocytogenes* a turgor of 14 atm (Tran et al., 2021) in chemically defined media of 0.23 Osm. This suggests that high turgor is a general feature of gram-positive bacteria. The primary focus of both studies was to measure diffusion of cytoplasmically expressed GFP, which should be reduced by osmotic shock as cytoplasmic volume is reduced. They found large reductions in D_L_ even for small osmotic shocks. This contradicts the lack of response for shocks less than 1 Osm reported in the earlier studies (Figure 4). This unexplained plateau for low osmolal shocks may not be a general feature of gram-positive bacteria, and should be reinvestigated.

### Cryosectioning EM Shows a Periplasm in Gram-Positive Bacteria; Tomographic cryoEM Does Not

**FIGURE 5:**
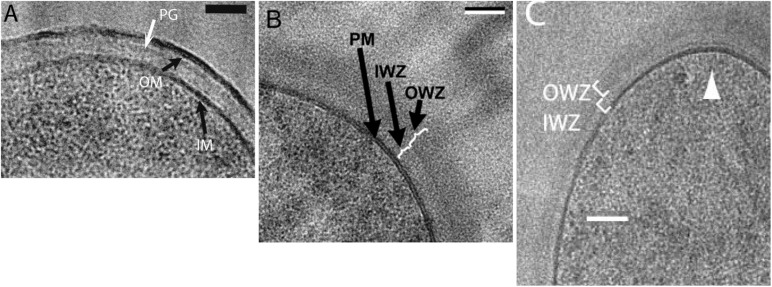
CryoEM using high pressure freezing and cryosectioning shows a periplasmic space in *E. coli* [**A**: (Matias et al., 2003)] and *B. subtilis* [**B**: (Matias and Beveridge, 2005) and **C**: (Zuber et al., 2006)]. PGW is indicated by PG in **(A)** and OWZ (outer wall zone) in **(B,C)**. The periplasm is indicated by IWZ (inner wall zone) in **(B,C)**. Reprinted from the referenced works with permission.

Some of the best evidence for a periplasm in bacteria is the cryoEM of Matias and Beveridge. This group initially imaged *E. coli*, where they could resolve the IM, the OM and a thin PGW. There was a clear periplasmic space of 11 nm separating the IM and PGW (Figure 5A; Matias et al., 2003). The space between the IM and OM corresponds to 13% of the cell volume, consistent with that measured by Cayley et al. (2000). The Beveridge group subsequently used the same cryosectioning technology to image a periplasmic space of 22 nm In *B. subtilis* (Figure 5B) and 16 nm in *S. aureus* (Matias and Beveridge, 2005, 2006). Another laboratory using the same cryosectioning technology obtained similar images of a periplasm in *B. subtilis* (Figure 5C) and other gram-positive bacteria (Zuber et al., 2006).

These results were contradicted by Beeby et al. (2013), who reported that most gram-positive species showed no periplasmic space. In their cryoEM images the CM was pressed against the PGW. However, these contradictory studies used different cryoEM techniques. The Beveridge group, which showed a periplasm in both gram-negative and gram-positive bacteria, used high pressure freezing followed by cryosectioning to image thin frozen sections. Beeby, Jensen and colleagues, who reported no periplasm for gram-positive bacteria, used plunge freezing, and they imaged whole bacteria through a series of tilts. They then used tomographic reconstruction to calculate the image of a thin section at the midplane of the cell. The different results might be due to the different technologies. Curiously, however, the Jensen group (Chang et al., 2016) obtained images of the *E. coli* periplasm that are almost identical to those of the Beveridge group.

A recent cryoEM study of *B. subtilis* also showed no periplasm (Khanna et al., 2020). Because *B. subtilis* is too large for direct imaging, this group used ion beam milling to etch the cells to a ∼200 nm section of the cell center. The tomographic reconstructions of these sections were of sufficient quality to image FtsA and FtsZ filaments beneath the cell membrane. Although not explicitly discussed the article, the images showed the ∼25 nm PG layer abutting directly the CM, with no periplasmic space.

This major discrepancy for cryoEM, where some studies see a periplasm in gram-positive bacteria, while others do not, remains unresolved.

### How Teichoic Acids Can Maintain the Osmolality of the Periplasm

As discussed above *E. coli* maintains the osmolality of the periplasm by OPGs, which are anionic glucose oligomers, ∼2,300 Da with an average –5 charge. Their neutralizing cations and associated Donnan equilibrium maintain an osmolality that matches that of the cytoplasm (Kennedy, 1982; Miller et al., 1986; Cayley et al., 2000). OPGs are retained in the periplasm because they are too large to pass through the small porins of the OM-PGW layer. OPGs would not work in gram-positive bacteria because they would easily pass through the more porous PGW. Note that I had earlier concluded that the PGW of gram-positive bacteria might have a limited porosity, blocking molecules larger than ∼1,200 Da (Erickson, 2017). However, my colleague Masaki Osawa later convinced me that the PGW is probably porous to globular molecules of 20,000 Da or more (Osawa and Erickson, 2018). In that case OPGs, and even longer glycan chains, would slide through the pores of the PGW and escape.

**FIGURE 6:**
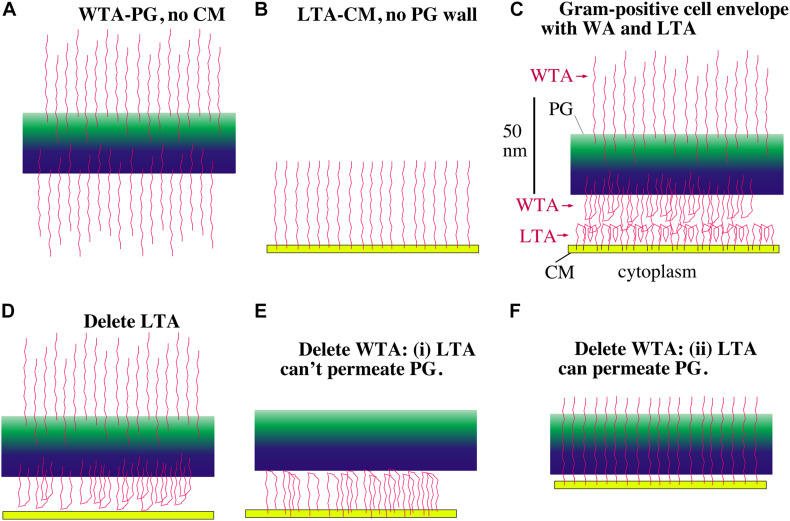
Models showing how LTA and WTA can maintain a periplasm. The PGW is green, the CM is yellow and the TAs are red. WTAs are thought to be longer than LTAs but are shown here the same length for drawing convenience. **(A)** Without a CM, the WTA would project straight out on both sides of the PG wall. **(B)** Without a PG wall, LTA would project from the CM. **(C)** In the gram-positive cell envelope the LTA and WTA in the periplasm will meet and compress each other until their electrostatic repulsion matches the turgor pressure of the cytoplasm. **(D)** If LTA is deleted, the WTA facing the CM can still support a periplasm, although the periplasmic width should be compressed. **(E,F)** If WTA is deleted there are two possibilities. If the LTA is blocked from penetrating the PG wall, it can still maintain a periplasm **(E)**. If the LTA can freely permeate the PG, however, it should not be able to maintain a periplasm **(F)**. Recent images of the periplasmic face of the PGW show a tight mesh of glycan strands that the LTA should not penetrate (Pasquina-Lemonche et al., 2020), relieving the concern of **(F)**.

There is, however, an alternative polyanion in gram-positive bacteria that is an excellent candidate for maintaining periplasmic osmolality – teichoic acids. Teichoic acids (TAs) are chains of ∼25 glycerol phosphates or ribitol phosphates, where the phosphates give them a polyanionic character. They exist in two forms. Lipoteichoic acid (LTA) has a terminal lipid that inserts into the periplasmic side of the CM. Wall teichoic acids (WTA) are covalently attached to the peptidoglycan. Although the TAs would easily pass through the PGW if they were free chains, the anchors to the CM or PGW prevent their escape and trap them in the periplasmic space (Figure 6).

Oku et al. (2009) have previously suggested that TAs may be functioning to maintain the osmolality of the gram-positive periplasm, similar to the role of OPG in gram-negative bacteria. They knocked out the *ltaS* gene in *S. aureus*, which completely eliminated LTA, but left WTA. The cells lacking LTA were viable but had a growth defect – they could grow at 30° but not 37°. If, however, the NaCl in the growth media was raised from 0.17 M to 1.3 M, or if sucrose was added to 1.1 M, cells lacking LTA could grow at 37°. Moreover, if the NaCl was decreased to 0.08 M, cells could not even grow at the permissive 30°. Thus cells lacking LTA are viable, but only in a high osmolal growth medium. Similarly in *B. subtilis*, Schirner et al. (2009) showed that knockout of LTA produced viable cells, but with defects in cell division and separation. They did not explore changing the osmolality of the growth media.

Previous studies showed that knockout of WTA produced mild growth defects in *S. aureus* (Weidenmaier et al., 2004), but caused severe cell rounding and clumping in *B. subtilis* (D’Elia et al., 2006). The double knockout was lethal in both *S. aureus* and *B. subtilis* (Oku et al., 2009; Schirner et al., 2009). Here we follow up the suggestion of Oku et al. (2009), that TAs may function to maintain the osmolality of the periplasm.

### LTA and WTA May Force the Existence of a Periplasmic Space

Figure 6 shows a schematic diagram for how the LTA and WTA chains could be arranged in the periplasm. The LTA chains are spaced about 2 nm apart, which is much closer than their 22 nm length (see the next section). This fits the classic description of a polymer brush. If an LTA chain were isolated in solution it would tend to collapse into a spherical blob. When grafted onto the CM at high density, the flexible chains are forced to extend into the brush. Several physical-chemical forces contribute to this extension: (1) excluded volume, where the chains cannot occupy the same space; (2) electrostatic repulsion within and between chains; (3) reduced entropy of the chains as they are confined to a narrow cylinder; (4) reduced entropy of the counterions as they are concentrated near the anionic charges. These forces not only cause the TA chains to extend into the brush, but they will generate a pressure when the brushes are pushed toward a surface or toward each other. These physical chemical forces can be used to calculate this pressure, as described by Pincus (1991) and by Zhulina and Rubinstein (2012). A detailed application of polyelectrolyte brush theory to TAs should be possible, but is beyond the scope of the present article.

Here I will develop two arguments to explain qualititatively how the TAs could generate a periplasmic space that balances turgor pressure. I will first present a pictorial description based on electrostatic repulsion of TAs. This is similar to the simple explanation of cartilage mechanics in histology texts. I will then develop a calculation of the fixed charge density of the TAs, which bring a concentration of neutralizing counterions approximately equal to the osmolality of the cytoplasm.

In the pictorial description, consider first the WTA chains. Figure 6A shows these negatively charged chains repelling each other and extending from both sides of their attachment to the PGW. Figure 6B shows LTAs, which are tethered to the periplasmic side of the CM, extending into the periplasmic space. The important diagram is Figure 6C, which shows how the WTA and LTA should interact in the full bacterial envelop. Since both WTAs and LTAs are long enough to extend the full 22 nm [according to Matias and Beveridge (2005)] width of the periplasm, and they are both negatively charged, they will repel each other. If there were no turgor pressure they would generate a periplasmic space equal to the sum of their lengths. If the CM is under turgor it should push the LTAs and WTAs toward each other, forcing them to interdigitate charges or fold back upon themselves (Figure 6C). The CM will be pushed by turgor toward the PGW until the electrostatic repulsive force of the WTA-LTA balances the turgor pressure.

Gene knockout studies in both *S. aureus* and *B. subtilis* have shown that one can eliminate either LTA or WTA but not both (Oku et al., 2009; Schirner et al., 2009). Let us explore how these knockouts would affect the periplasm in our model. If LTA is eliminated the WTA would project equally inward and outward. Those WTA chains projecting inward can’t penetrate the CM, so they would push against it, establishing a periplasm (Figure 6D). (Although we have ignored it for the model, WTA are up to twice as long as LTA (Neuhaus and Baddiley, 2003; Brown et al., 2013), so they would have to fold back even without repulsion by LTA.) The CM would press against the WTA compressing them until the electrostatic repulsion balanced the turgor pressure. The periplasm should be thinner than with both WTA and LTA because the density of negative charges is lower, but it should still exist.

Eliminating WTA seemed at first more problematic for the simple model. There would be no problem if the LTA were blocked from penetrating the PGW (Figure 6E), because the negative charges of the LTA would then be confined to the periplasmic space. However, if the PGW is porous (Osawa and Erickson, 2018), the LTA chains may be able to extend through the PGW, and would not establish a periplasm (Figure 6F). This concern now seems to be relieved by a recent study imaging the PGW by high resolution atomic force microscopy (Pasquina-Lemonche et al., 2020). The periplasmic surface of the PGW appeared to be a dense mesh of glycan strands, with pores noted up to a maximum diameter of 6.4 nm, although most pores were smaller. The authors did not analyze this inner surface in detail, but I counted roughly 30 pores over a 60 nm square in their Figure 2F. Using the value of 5.4 nm^2^ per LTA calculated below, there would be 667 LTA in this same 60 nm square. Thus, there is only one pore for twenty LTAs. Inserting a single LTA into a pore would be inhibited by its loss of entropy; trying to fit two LTAs into a single pore would encounter additional electrostatic repulsion. Overall, the inner surface of the PGW would seem to be impenetrable by LTAs, negating the concern of Figure 6F.

### The Counterions That Neutralize LTA and WTA Could Approximately Counter the Cytoplasmic Osmotic Pressure

We now turn from the pictorial description of polyanionic TA chains repelling each other, to a more quantitative description based on fixed charge density. This is similar to the quantitative description of cartilage mechanics (Lu and Mow, 2008). LTAs are very abundant, being 1/9 (Percy and Grundling, 2014) or 1/5 to 1/10 (Oku et al., 2009) of the outer leaflet lipids. The area per lipid in a bilayer is generally given as 0.6 nm^2^. If 1/9 of lipids are LTA, the area per LTA is 5.4 nm^2^. A typical LTA is a chain of 25 GroP (Percy and Grundling, 2014), which should be fully extended because the negative charges will repel each other. At 0.9 nm per GroP, the extended LTA chains would be 22 nm long, which is equal to the width of the periplasm measured by cryoEM (Matias and Beveridge, 2005).

The phosphate groups in TAs are in a diester linkage and carry a single minus charge. We can calculate the effective concentration of the P^–^ as follows. The volume per LTA is 5.4 nm^2^ × 22 nm = 119 nm^3^. The 25 P^–^ in 119 nm^3^, gives 0.21 nm^3^ per P^–^, which converts to a concentration of 0.35 M.

WTAs are comparable in number and density to LTAs (Neuhaus and Baddiley, 2003); 1 out of 9 MurNac in the PGW has an attached WTA (Brown et al., 2013). WTAs are twice as long as LTAs (Neuhaus and Baddiley, 2003), but only half are projecting into the periplasm (Figure 6), so we can assume the WTA contribute a concentration of P^–^ approximately equal to the 0.35 M of the LTA, for a total of 0.7 M. It is important to note, however, that 20–70% of the glycerol phosphate groups have an attached D-alanyl group (Neuhaus and Baddiley, 2003; Percy and Grundling, 2014). Each D-alanyl creates a positive charge, which effectively neutralizes one P^–^. The D-alanyl groups are dynamically removed and re-added (Neuhaus and Baddiley, 2003), providing a mechanism to modify and fine tune the anionic concentration of the periplasm. Perego et al. (1995) measured the percent D-alanylation for *B. subtilis* growing in a defined medium of ∼0.3 Osm. 44% of the glycerophosphate groups in LTA and 9% in WTA were D-alanylated, for an average 27%. The net concentration of anionic P^–^ in the periplasm will then be ∼0.5 M. This will bring into the periplasm a concentration of 0.5 M Na^+^ counterions.

A 19 atm turgor pressure means that the cytoplasm has an excess osmolality of 0.75 Osm relative to the growth medium. An isoosmolar periplasm should therefore be 0.75 Osm above the growth medium. The P^–^ groups themselves will contribute little to the osmolality of the periplasm because 25 of them are connected into a single TA chain. The osmolality will be generated primarily by the 0.5 M Na^+^ cations that neutralize the P^–^. The Donnan equilibrium will increase periplasmic osmolality only slightly in normal growth medium. Using the formulation of Tsujii (2002) the Donnan equilibrium will add 0.024 M to the periplasmic osmolality for growth medium containing 0.1 M NaCl (e.g., LB with 0.5% NaCl). The Donnan contribution will rise to 0.25 M in 0.4 M NaCl, a very high-salt medium. The anionic surfaces of membrane proteins should contribute additional neutralizing Na^+^. The 0.5 M Na^+^ was a very approximate calculation. Overall, the cations neutralizing the anionic TAs and proteins should approximately match the excess osmolality of the cytoplasm, leaving the periplasm and cytoplasm isoosmotic.

We should recognize that the simple calculation of counterion concentration is only one contribution to pressure in the brush theory. The situation is especially complicated when the NaCl in the growth media approaches or exceeds the fixed charge density of the TAs. However, normal growth media contain 0.5% or 1% NaCl (0.08 M or 0.17 M) well below the 0.5 M concentration of TA anions. This corresponds to Regime I of Zhulina and Rubinstein (2012), where the osmotic pressure is determined primarily by the concentration of counterions. Thus, in normal growth media the concentration of counterions is a good approximation to the osmolality of the periplasm.

### Comparing the Periplasm to Articular Cartilage

The description above of TAs acting through electronegative repulsion was inspired by the treatment of articular cartilage presented in many textbooks. Articular cartilage is a rigid tissue covering the surfaces of bones where they meet in a joint. Articular cartilage is a compressible cushion that can support very high compressive forces, many times body weight, up to 20 atm at the hip (Lu and Mow, 2008). The chemical basis for this rigidity to compression is a gel of polyanionic glycosaminoglycans, which are chains of sugars with negatively charged COO^–^ and SO_3_^–^ groups attached. The glycosaminoglycans are attached to other molecules to make proteoglycan aggregates, which reach a length of ∼40 μm. The glycosaminoglycan chains are highly concentrated, and electrostatic repulsion would drive them to escape. They are held in the concentrated state by a series of strong chemical bonds within the proteoglycan aggregate. The proteoglycan aggregates themselves are retained in the cartilage matrix by physical entrapment as they snake through a network of collagen fibrils (Lu and Mow, 2008). The rigidity to compression can be attributed pictorially to the repulsion of the concentrated, polyanionic glycosaminoglycan strands.

A more quantitative analysis of cartilage mechanics is based on the fixed charge density, which has been reported to be 0.28 M (Lesperance et al., 1992) or 0.4 M (Ehrlich et al., 1998). This is somewhat less than the 0.5 M fixed charge density estimated above for the combined LTA and WTA in the gram-positive periplasm. This analogy suggests that the periplasm may be considered a gel with a rigidity similar to that of articular cartilage. This rigid gel is pressed against the PGW on the outside, and the CM presses against it on the inside. The TA gel thus supports the turgor pressure of the CM. Note that in spite of its mechanical rigidity, articular cartilage permits diffusion of ions and protein molecules. The same would be true of a periplasm with highly compressed TAs. These should permit the functioning of PGW remodeling enzymes and diffusion of their substrates.

## Conclusion

I have presented arguments that a periplasmic space is needed for the PGW synthesis proteins to function, and I developed a quantitative estimate that TAs could generate a concentration of counterions that approximately balances the osmolality of the cytoplasm. This would leave the CM floating between the turgor pressure of the cytoplasm and the brush pressure of the TAs in the periplasm, and subject to no net force from turgor. As noted above, a full treatment by brush theory is needed to complete this argument, especially for growth in high salt media. There are also major experimental gaps that need to be filled. The failure of cryoEM tomography to image a periplasm is a concern that needs to be resolved by electron microscopists. Most important would be to repeat for gram-positive bacteria the isotopic labeling experiments of Stock et al. {1977 #10230} and Cayley et al. {2000 #10236} to establish the existence and osmolality of the gram-positive periplasm. Alternatively, the periplasm could be imaged by super-resolution light microscopy to quantitate its response to osmotic shock, as has been done for *E. coli* (Pilizota and Shaevitz, 2013).

Although septation may not need to overcome turgor pressure, force is needed to bend the membrane and perhaps to invaginate into the restricted volume of the cytoplasm. There are currently three favored mechanisms for generating this force. First is FtsZ pulling on the CM from the inside. This mechanism is supported by *in vitro* experiments showing that FtsZ alone can constrict liposomes (Osawa et al., 2008; Osawa and Erickson, 2013); also FtsZ is apparently needed to initiate constriction *in vivo* (Monteiro et al., 2018; Whitley et al., 2021). The second potential force mechanism is PGW synthesis pushing the CM from the outside. Supporting this is the observation that mutations in FtsI reduced the rate of constriction (Coltharp et al., 2016); however, this would also occur if PGW synthesis was simply limiting constriction, rather than generating the force. The third force mechanism is excess membrane production (Osawa and Erickson, 2018). Once a constriction furrow has been initiated, excess membrane production would preferentially add to this constriction as opposed to initiating a new invagination. In support of this, experiments with L forms have shown that excess membrane production is crucial to their division (Mercier et al., 2013).

In *Streptoccous pneumoni*ae and *B. subtilis*, when FtsZ treadmilling was blocked by the drug PC190723, Z rings that had already initiated constriction could continue constricting to complete division (Monteiro et al., 2018; Whitley et al., 2021). This suggests that, after constriction is initiated by FtsZ, the primary forces are the second and/or third mechanisms. It is difficult to separate the roles of these two mechanisms since they are likely physically linked. A simple scenario would have FtsZ bending the membrane to initiate constriction, with excess membrane production forcing the continued invagination, and PGW synthesis limiting the rate of constriction and perhaps contributing to the force.

### An Analogy for Cell Division Needing to Fight Turgor or Not

Imagine a space station capsule where the outer wall is a strong, airtight fabric, forming an elongated cylinder that contains the 1 atm pressure. Suppose the Russians and Americans had a serious dispute and decided to split the capsule in two. One engineer proposed constructing a large belt that could wrap around the cylinder at mid-length, and using a large winch to gradually tighten the belt to squeeze the cylinder in two. Another engineer suggested that this winch and belt might not be able to achieve the necessary force with the energy reserves available. She proposed that they use their store of excess fabric to build two closely parallel walls, each securely glued to the outside cylinder. When the walls were complete she proposed a space walk to cut the cylinder between them. Since the astronauts are in zero gravity, only negligible force would be needed for any step.

## Data Availability Statement

The original contributions presented in the study are included in the article/supplementary material, further inquiries can be directed to the corresponding author.

## Author Contributions

HPE did the analysis and wrote the manuscript.

## Conflict of Interest

The author declares that the research was conducted in the absence of any commercial or financial relationships that could be construed as a potential conflict of interest.
